# A new flavor of synthetic yeast communities sees the light

**DOI:** 10.1128/mbio.02008-23

**Published:** 2025-02-06

**Authors:** Vicente Rojas, Daniela Rivera, Carlos Ruiz, Luis F. Larrondo

**Affiliations:** 1 ANID-Millennium Science Initiative Program—Millennium Institute for Integrative Biology (iBio), Santiago, Chile; 2 Facultad de Ciencias Biológicas, Pontificia Universidad Católica de Chile 28033 , Santiago, Chile; 3 Centro Científico y Tecnológico de Excelencia Ciencia & Vida, Fundación Ciencia & Vida, Huechuraba, Santiago, Chile; Instituto Carlos Chagas, Curitiba, Brazil

**Keywords:** yeast, synthetic ecology, optogenetics, optoecology, synthetic biology, *Saccharomyces cerevisiae*

## Abstract

No organism is an island: organisms of varying taxonomic complexity, including genetic variants of a single species, can coexist in particular niches, cooperating for survival while simultaneously competing for environmental resources. In recent years, synthetic biology strategies have witnessed a surge of efforts focused on creating artificial microbial communities to tackle pressing questions about the complexity of natural systems and the interactions that underpin them. These engineered ecosystems depend on the number and nature of their members, allowing complex cell communication designs to recreate and create diverse interactions of interest. Due to its experimental simplicity, the budding yeast *Saccharomyces cerevisiae* has been harnessed to establish a mixture of varied cell populations with the potential to explore synthetic ecology, metabolic bioprocessing, biosensing, and pattern formation. Indeed, engineered yeast communities enable advanced molecule detection dynamics and logic operations. Here, we present a concise overview of the state-of-the-art, highlighting examples that exploit optogenetics to manipulate, through light stimulation, key yeast phenotypes at the community level, with unprecedented spatial and temporal regulation. Hence, we envision a bright future where the application of optogenetic approaches in synthetic communities (optoecology) illuminates the intricate dynamics of complex ecosystems and drives innovations in metabolic engineering strategies.

## INTRODUCTION

### Life in microbial communities: a daily challenge

The Earth is populated by a huge diversity of organisms, which rarely exist in isolation. Instead, they constantly interact with other biological agents shaping ecosystems and occupying distinct habitats (1, 2). Often, large populations of these individuals share ecological niches, forming communities of varying complexity. Classic examples of microorganism-based communities include lichens (3, 4), mycorrhiza (5–7), bacterial biofilms (8, 9), and human intestinal flora (10, 11). While natural microbial ecosystems typically consist of diverse organisms from various taxa, they may also include mixtures of strains within a single species. Intraspecies consortia, although less versatile in their observed functions, are considerably easier to maintain and study in laboratory settings (12). Given that environments are subject to constant fluctuations, community members must exhibit flexibility to achieve equilibrium, where the dynamics of cooperation and competition enable effective nutrient acquisition and sustain trophic and metabolic relationships (13). Nevertheless, shifts in community composition can be challenging, and community collapse may occur in the face of extreme conditions. Thus, the proportion of each strain/organism/taxa, along with their respective metabolic capabilities, is relevant to sustain the consortia over time (14, 15).

### Microorganisms can provide a macro perspective

**Fig 1:**
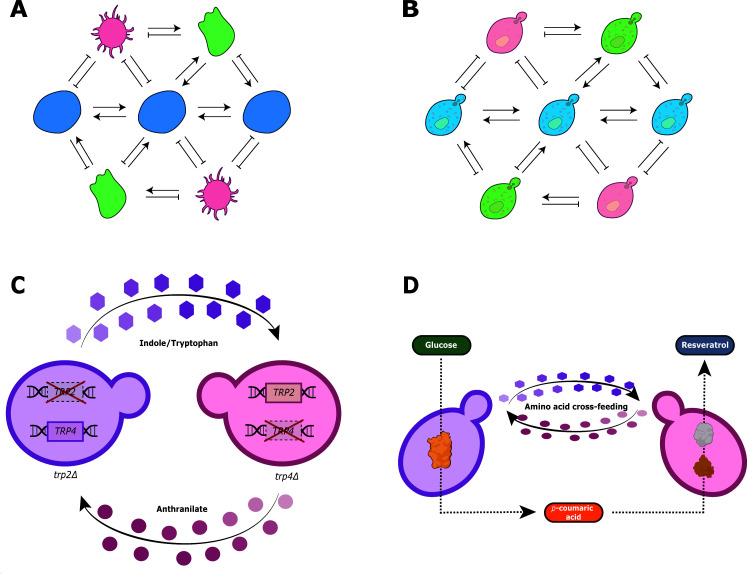
Synthetic microbial communities and their applications. Synthetic microbial communities are user-manipulated systems in which distinct species or variants of the same organism interact dynamically to form defined and controllable ecosystems (**A**). These communities allow researchers to study ecological and evolutionary processes under controlled conditions, using microbial models such as *Saccharomyces cerevisiae*, where different strains or variants, possessing particular metabolic or signaling modules, can interact in diverse ways (**B**). Consortia involving distinct mutant *S. cerevisiae* strains have been engineered to study ecological dynamics driven by nutritional co-dependence (**C**). This approach has also been utilized to produce and enhance valuable biomolecules, such as resveratrol, by distributing biosynthetic pathways among community members (**D**). Panels C and D are adaptations of the original figures from Aulakh et al. (30) and Peng et al. (31), respectively. Throughout this text, we will use the term “species” according to its classic definition: organisms that share high genetic similarity (as determined by phylogenetic markers) and exhibit consistent ecological roles. In contrast, “variants” will refer to subpopulation within a species that display subtle genetic or functional differences, shaping their adaptation and diversification in ecological interactions.

Due to desirable features such as tractable genetics, fast life cycles, and the ease of growth requirements, artificial communities based on model microbes have garnered significant attention, driving the development of bold synthetic biology strategies (Fig. 1A) (16). Indeed, in order to study processes at intercellular levels, artificial consortia involving bacterial-bacterial, fungal-fungal, or bacterial-fungal interactions have been successfully designed and implemented. Here, microorganisms compete for scarce nutrients and limited space, causing the activation of several genetic programs allowing them to overgrow and eliminate potential competitors, including hoarding of micronutrients, optimization of their metabolism, and exploitation of nutrients produced by other cells (17). However, recent evidence, obtained with synthetic communities, exemplifies how competition can progressively decrease due to selective forces and ecological conditions, leading to stable co-existence (18). Importantly, engineered ecosystems allow programming division of labor across community members, performing more intricate functions than clonal populations (19, 20). This approach refers to the physical separation of biological activities between engineered strains/organisms to optimize the performance of individuals and, therefore, of the entire community. Furthermore, through this strategy, synthetic communities overcome the limitations found in all-in-one highly modified strains, such as the metabolic burden caused by pathway engineering or toxicity caused by the produced molecules (21). By dividing these challenges within the community participants, they emulate the efficiency observed in natural consortia, reducing engineering complexity and enabling high-efficiency production of molecules of interest. Therefore, it is possible to implement highly intricate intercellular networks, expanding the limits of synthetic circuits and metabolic pathways (22). The compartmentalization of pathway components between interconnected cell groups provides other positive effects for artificial multicellular systems, such as the increase of genetic stability and the passive filtering of transcriptional noise (23). Interestingly, it has been demonstrated that resource sharing between community members can promote the spontaneous appearance of division of labor (24). To ensure connectivity, the secretion of diffusible signaling molecules serves as a communication mechanism enabling the modulation of biological outputs, where each member could play a vital role in consortia functioning (25). However, it is also likely to create ecosystems with unbalanced composition, where a small subset of members serves as the core by harboring the essential genetic modules needed to sustain both themselves and the rest of the much larger population, which in turn can contribute with additional functions extending the versatility of the whole system (26). Notably, a battery of experimental methodologies and computational approaches allows the analysis of microbial consortia in terms of critical parameters such as population ratios, single-cell dynamics, growth rates, and identification of shared metabolites (27–29). Hence, studying microbial communities is tremendously helpful in expanding our current knowledge about the plasticity of organisms and their respective ecological interactions under different contexts, as well as for engineering improved consortia to address more complex processes.

## NOVEL WAYS OF ADDRESSING COMPLEXITY

### Yeast in modular and dynamic synthetic communities

Synthetic microbial communities are modular—yet dynamic—since they can perform different processes depending on their members and the corresponding communication modes (32). While user-defined consortia may comprise multiple intra- and interkingdom interactions, this review mainly focuses on those containing the budding yeast *Saccharomyces cerevisiae*. The latter is a unicellular fungus from the Ascomycete division, whose 12 Mbp genome (distributed in 16 chromosomes) contains about 6,000 genes (33, 34). Its high rate of homologous recombination, a doubling time of 1.5 h, the easiness of growing it on different carbon and nitrogen sources, and the constant development of tools for its genetic edition have positioned *S. cerevisiae* as a premier model organism to address questions going from molecules up to ecosystems (Fig. 1B). The biochemical and cellular studies in budding yeast have helped to unveil the bases of eukaryotic biology (35). In addition, *S. cerevisiae* has historically been a relevant microorganism exploited for thousands of years to elaborate bread and obtain alcoholic beverages due to its outstanding fermentation capacity (36). For these and many more reasons, consortia involving yeast have great potential to leverage, develop, and exploit various dimensions of fundamental and applied aspects of synthetic communities. Below, we summarize some fascinating examples of artificial communities involving *S. cerevisiae*.

### Emulating nature at the laboratory level

Synthetic ecology seeks to recreate natural interactions between organisms in specific niches (37). Using well-characterized cell models and controlled lab conditions, it is possible to study and challenge the interaction dynamics in mixtures of diverse populations (38). In this context, microbial communities often display an extensive metabolite exchange, which plays a pivotal role in shaping growth dynamics within defined ecosystems (39). For instance, in *S. cerevisiae* colonies, evidence suggests that a variety of molecules are actively imported and exported, contributing to the establishment of a substantial extracellular resource pool. These shared metabolites can significantly impact community stability and foster cooperative interactions, which are crucial for maintaining the overall functionality and resilience of the microbial consortium (39). Yeast prototroph cells preferentially take up essential metabolites from the medium when available, rather than activating the corresponding biosynthetic pathways (39). In a pioneer study, engineered yeast strains were evaluated in co-cultures as well as monocultures (40). Cell mutants on either *ADE8* or *LYS2* genes are unable to produce adenine and lysine, respectively; however, they were also modified to overproduce (without feedback inhibition) the metabolite missing in the other strain. Thus, when strains were co-inoculated in a selective medium, the cell density of each strain increased as a function of time due to cooperative behavior (40). The latter, also known as syntrophy, occurs when co-cultured organisms experience an obligate mutualism by sharing one or more metabolic intermediates, and the proliferation of both types of cells depends on the substrate provided by the other one. As expected, the growth of mutants was not observed when they were individually evaluated in a medium lacking adenine and lysine (40). A similar approach was used to evaluate the community behavior by varying the proportion of overproducer mutant strains (41). Notably, symmetric and asymmetric co-cultures did not show collapse. Furthermore, denser communities were established by adding different clones of each strain type to extend beyond pairwise interactions. Thus, four-, six-, and eight-member consortia involving functionally redundant strains displayed normal growth in a selective medium. Interestingly, this increase in diversity richness allowed high levels of persistence when the ecosystems were challenged by including exploiter cells (41). In a recent work, a high-throughput analysis of co-cultures involving yeast auxotrophic mutants was carried out, where a specific pair of strains also led to the spontaneous establishment of a community by metabolic co-dependence (30). Although multiple combinations failed to compensate for each other nutritional deficiencies in co-cultures, mutants of the *TRP2* and *TRP4* genes were able to grow by exchanging intermediaries of the tryptophan biosynthetic pathway such as anthranilate and indole (Fig. 1C) (30). Similarly, another study developed a toolkit involving donor and receiver yeast cells that can establish stable co-cultures by exchanging different essential amino acids such adenine, lysine, and tryptophan (31). By manipulating metabolite production rate, initial population ratio, metabolite supplementation, and initial cell density, some essential variables, such as growth rate, size, and composition of two- and three-member consortia, were finely controlled (31), which highlights the modularity and versatility of this synthetic yeast syntrophic consortia.

Moreover, interspecies yeast co-cultures have been implemented to unveil how yeast species interact in wine elaboration (42). Thus, *S. cerevisiae* strains were inoculated into synthetic must in pairwise combinations with non-*Saccharomyces* yeasts, including *Hanseniaspora uvarum*, *Lachancea thermotolerans*, *Starmerella bacillaris*, and *Torulaspora delbrueckii*. By analyzing growth curve kinetics, co-cultures showed positive and negative outcomes regarding monocultures. Interestingly, the characteristics of each user-defined community are the result of interactions at the species level as well as at the strain level (42). In a more intricate design, yeast cells have been combined with cells from other kingdoms. Indeed, *S. cerevisiae*’s ability to produce acetaldehyde was cleverly exploited by using the budding yeast as a molecule sender, allowing the assessment of interkingdom parasitism (43). Thus, an inducible third-party paradigm was tested: in the presence of acetaldehyde, mammalian cells secreted β-lactamase allowing *Escherichia coli* to survive in the ampicillin-containing medium. High bacteria levels affected the growth of the mammalian cell line, but in a dual system lacking yeast, the mammalian cells easily predominated over *E. coli* as no β-lactamase was produced. Nevertheless, when yeast was present, conditional parasitism was established, allowing oscillations in the levels of the mammalian and bacterial cell numbers as the ongoing molecular communication modulated mutual population size (43). Such an example shows how *S. cerevisiae* can be used in synthetic ecology, revealing an interspecies interaction that emulates natural oscillating parasite-host or predator-prey behaviors.

### Dividing to conquer: improving cell factories

Importantly, *S. cerevisiae* is one of the most extensively used hosts to produce high-value metabolites at the biotech level (44), and not surprisingly, emerging approaches have actively focused on further optimizing metabolic bioprocessing to enhance the industrial production of high-value compounds such as pharmaceuticals and fuels. Genetically modified yeast strains offer a more cost-effective and sustainable alternative to traditional chemical synthesis or extraction from natural sources (45). Relevantly, engineered microbial consortia often show enhanced performance in the generation of commercial products compared to autonomous single-cell systems (46). As hinted earlier, a well-established community, with explicit cellular communication among its members, can be engineered to strategically and efficiently divide biosynthetic pathways, thereby maximizing the yield of a desired product (47). Moreover, the division of labor not only enhances metabolic outcomes by splitting the engineered system but also by reducing byproduct formation by enabling upcycling of otherwise undesired compounds, thereby improving final product yield (48). When the rational implementation involves sequential activation of the different members, the synthetic ecosystem could resemble an assembly line to predictably obtain the biological output (49). Thus, a yeast system, based on the syntrophic behavior of tryptophan auxotrophs, was successfully coupled to the production of a metabolite of interest such as malonic semialdehyde (30). After splitting the corresponding biosynthetic pathway between the two strains, the molecule of interest was obtained in higher concentrations compared to single cells harboring all the enzymes (30).

A follow-up study, building on the characterization of cross-feeding behavior in *S. cerevisiae trp2*Δ *and trp4*Δ mutants (30), explored similar interactions in the equivalent *Yarrowia lipolytica* mutants, confirming that syntrophic co-cultures can establish intraspecies cross-feeding within this yeast (21). Moreover, co-culturing the *Y. lipolytica* with the *S. cerevisiae* auxotrophic mutants enabled interspecies syntrophy, resulting in enhanced growth compared to monocultures through mutual exchange of essential metabolites. Notably, the authors leveraged this interspecies cross-feeding to boost the bioproduction of 3-hydroxypropionic acid (3-HP). By establishing a division of labor in the 3-HP biosynthetic pathway between both strains, they achieved higher yields of the compound compared to monocultures. These findings underscore the potential of syntrophic communities to address challenges such as metabolic burden and toxicity in bioproduction processes (21). Division of labor in *S. cerevisiae* has also been carried out to develop a cellulosome (50, 51). Here, three strains were engineered to secrete enzymes related to cellulose degradation. A fourth strain was modified to superficially express a scaffold protein, where the extracellular proteins could couple through dockerins to increase the substrate conversion into simpler sugars, which in turn are transformed into ethanol with high yield (50, 51). Also related to fermentative processes, *S. cerevisiae* strains have been genetically modified to behave as glucose- and xylose-utilizing specialists (52). Such strains were obtained and evaluated in a medium containing a mixture of sugars. By adjusting the initial composition and the timing of inoculation, the division of labor allowed precise control of the fermentation dynamics (52). Similarly, engineered specialist strains were used to establish a stable three-member consortium with the capacity to ferment a culture medium containing glucose, xylose, and arabinose (53). Interestingly, additional intraspecies communities have been established to produce valuable flavonoids, where the separation of enzymatic reactions between yeast strains led to improved performance in terms of final metabolite concentration and higher cell growth compared to single populations (54, 55).

Similarly, other studies have provided compelling proof of concept for modulating the expression of complex and antagonistic molecules, such as yeast strains engineered to produce insulin and glucagon in response to sugar inputs (56). By implementing antagonistic genetic modules to produce a diffusible signaling molecule, a community of both yeast strains can activate insulin production while repressing glucagon biosynthesis under high glucose concentrations. The opposite behavior was observed at low glucose concentrations, demonstrating that the consortium can fully adapt its dynamics in response to environmental perturbations (56). In addition, synthetic yeast co-cultures have also been used to produce a fragrance like raspberry ketone. By dividing the endogenous and exogenous biosynthetic genes into two or three strains, this valued aromatic compound varied its concentration depending on growth conditions, including the number and nature of the ecosystem members or their initial ratio and culture media (57). Likewise, based on the aforementioned toolkit involving donor and receiver strains, a yeast consortium was implemented to obtain another metabolite of commercial interest such as the antioxidant resveratrol by using glucose as substrate and *p-*coumaric acid as a key intermediate (31). Thus, the complete biosynthetic pathway was separated into two modules among mutant strains exhibiting nutritional cross feeding (Fig. 1D) (31).

Synthetic communities involving different non-traditional yeast species have also been reported for bioprocessing strategies. Bioethanol, a renewable alternative powering the future of sustainable energy, has been produced from plant biomass through inoculation of *S. cerevisiae* and different yeast species, including *Pichia pastoris*, *Scheffersomyces stipites*, *Pichia angophorae*, *Spathaspora passalidarum*, *Geotrichum candidum*, and *Pichia barkeri* plus *Candida intermedia* in a three-member consortium (58–63). On the other hand, a consortium involving *S. cerevisiae* and other *Saccharomyces* species (*S. eubayanus* and *S. uvarum*) displayed a significant increase of indolic compounds in a wine fermentation context (64). Also, co-cultures between *S. cerevisiae* and *Y. lipolytica* have been developed to improve biodiesel production, where the mixture of both types of yeasts allows the simultaneous generation of critical precursors such as ethanol and fatty acids (65). Importantly, such experiences have mainly focused on naïve strains, with little genetic customization, and therefore, there is a lot of room for improvement and optimization, particularly as metabolic (i.e., syntrophy) strategies are implemented. For instance, a binary *Y. lipolytica* community has been reported as a platform for enhancing β-carotene production through the upcycling of a metabolic byproduct, such as citric acid, by a division of labor in carbon sources utilization (48). This system employs a wild-type strain and an upcycling strain, both engineered to integrate the β-carotene biosynthesis pathway. The wild-type strain metabolizes glucose as its primary carbon source, producing biomass and citric acid, whereas the upcycling strain utilizes citric acid produced by the first strain fueling it into β-carotene biosynthesis. By employing different media, such as minimal medium and lignocellulosic hydrolysates (e.g., urban pruning hydrolysates), alongside adjustments of the carbon-to-nitrogen ratio, the authors achieved a β-carotene yield of 0.11 g/g glucose in minimal medium. This yield amply surpasses previously reported single-strain effectiveness, which ranges from 0.003 to 0.05 g/g of β-carotene/glucose, demonstrating that synthetic communities significantly enhanced the production of this valuable compound. Thus, the resulting synthetic co-culture system doubled both biomass growth and β-carotene production compared to monocultures, underscoring the potential of co-cultures and byproduct recycling to advance more sustainable and efficient bioproduction pipelines (48).

Unsurprisingly, metabolic engineering has also explored communities that combine *S. cerevisiae* and prokaryotic cells. This approach has successfully harnessed the unique capabilities of each consortium member to produce a wide variety of high-value molecules (Table 1). Therefore, these examples illustrate how yeast has been extensively utilized, and many times heavily engineered to implement synthetic consortia for industrial purposes, expanding the possibilities of producing valuable molecules of different natures.

**TABLE 1:** Interkingdom communities involving *S. cerevisiae* and bacterial cells for high-value metabolite production

Partner	Molecule of interest	Reference
*E. coli*	Flavonoids	(66, 67)
*E. coli*	Medicinal compounds	(68–70)
*E. coli*	Betaxanthins	(71)
*E. coli*	Bioethanol	(72, 73)
*Pseudomonas putida*	Polyhydroxyalkanoates	(74)
*Clostridium phytofermentans*	Bioethanol	(75)
*Actinotalea fermentans*	Methyl halides	(76)

### Yeast communities as biosensing powerhouses

Biosensing is a rapidly expanding research area enabling the detection of organisms and diffusible molecules (77). *S. cerevisiae* can naturally sense pheromone gradients, a crucial feature for its sexual reproduction. In response to α- or a-factor, large-scale changes in morphology, cell cycle, and gene expression are elicited (78, 79). Interestingly, synthetic communication circuits exploiting the mating pathway have also been developed. For instance, a three-member system was implemented to measure α-factor secretion by monitoring a reporter strain expressing a fluorescent protein gene (80). In this setup, the pheromone was produced by two additional strains: one secreted it constitutively, while the other utilized a synthetic genetic module to produce the signaling molecule in response to its own activity. The efficiency of communal information propagation was evaluated in liquid and immobilized media, revealing that the initial strain ratio significantly influences the response level, as one cell type functions as a signal amplifier (80). Additional yeast pheromone biosensors have been extensively tested in a community format, combined with inducible producer cells. In that context, an artificial intercellular system was reported by co-incubating two different *S. cerevisiae* populations. Yeast strains were engineered so that one could produce α-factor in a doxycycline-dependent manner and respond to it, while the other was designed solely to respond to the pheromone (81). Interestingly, a high degree of self-communication was observed at low initial cell density and high concentration of the exogenous inducer. Conversely, the community displays a high degree of neighbor communication at high cell density and high inducer concentration. Therefore, the consortium’s capacity to establish social or asocial behavior depends on previously user-defined parameters, and the response of the synthetic ecosystem can even be bimodal if the circuit complexity is extended by adding positive feedback and a signal degradation module (81). Conversely, sender strains have been engineered to regulate the secretion of α-factor from *S. cerevisiae* (αSc) in response to a battery of stimuli, including glucose, galactose, and sodium chloride (82). Additionally, reporter strains were designed to activate the transcription of a reporter gene upon detecting αSc, with modulation by 17-β-estradiol or a specific inhibitor of a mating kinase such as 6a. Several simple logic gates were successfully implemented by co-culturing the cells in different ways, including N-IMPLIES, IDENTITY, AND, NOT, NOR, OR, and NAND (82), and the yeast intercellular networks were evaluated in more complex Boolean circuits by introducing a second communication molecule such as α-factor from *Candida albicans*. Thus, a broad range of responses was evaluated in four- and five-member consortia (82). Interestingly, a follow-up study extended the strain repertoire by adding a genetic response to hormones such as dexamethasone, progesterone, and aldosterone (83). By testing many cell combinations, synthetic communities were simultaneously exposed to three, four, and even six inputs (83). On the other hand, yeast cells have been modified at a genetic level to implement artificial communities where interactions depend on a heterologous signaling molecule such as the cytokinin isopentenyladenine (84). In order to do this, a first strain was obtained to produce the plant molecule by incorporating the isopentenyltransferase gene from *Arabidopsis thaliana*. Similarly, a second strain was developed by coupling the isopentenyladenine receptor to a native intracellular pathway that induces transcriptional reporter expression to evaluate the biosensing capacity. As expected, the fluorescence intensity increased as the distance between both strains is reduced (84). A control experiment consisted of generating a single strain with the capacity to secrete and sense the same molecule. Interestingly, the response depended on the cell density, resembling a quorum-sensing behavior (84). Quorum-sensing mechanisms have been described in different microorganisms, where relevant biological processes such as virulence or motility are coordinately triggered in the entire population when a given signaling molecule reaches a specific concentration threshold (85, 86). The same behavior has been observed in synthetic systems designed to produce and respond to yeast pheromone upon tryptophan supplementation (87, 88). Furthermore, one of the most important effects of α-factor over *MATa* cells is the arrest at the G1 stage, guaranteeing that mating occurs between cells with the same ploidy. These features enable the rational design of synthetic consortia to manipulate signaling molecules, controlling member growth and community composition (89). Yeast cells have also been engineered to sense and secrete another plant hormone such as the auxin indole-3-acetic acid (IAA). In that study, strains responding to IAA, αSc, and β-estradiol were mixed in different combinations to produce direct reporter expression as well as signal amplification or attenuation (90). Thus, two-, three-, and four-member consortia were successfully implemented, allowing a variety of robust behaviors (90). Hence, these examples illustrate how *S. cerevisiae*, either as individual cells or within a community, can be extensively engineered to detect diverse molecules with varying kinetics and elicit distinct biological responses—features that can be harnessed for a wide range of biotechnological applications.

## OPTOECOLOGY: ILLUMINATING COMMUNITY INTERACTIONS

### Light enters the equation

Synthetic microbial communities are usually designed to yield informative and compelling phenotypes (13, 32), but precise control of community members and functions remains a key challenge. Robust ecosystems can be obtained only when the behavior of constituent members is accurately manipulated (38), and therefore, triggering phenomena of interest with high spatiotemporal resolution are strongly desirable. On the other hand, besides adding a new level of complexity, tunable artificial consortia can optimize the overall performance of systems with stochastic or constant activity. Furthermore, the use of inducers also allows the tuning of critical parameters, modulating the community composition. By exploiting the natural capacity of photoreceptors to perceive wavelength-specific photons, optogenetics has extensively been utilized to modulate biological properties with striking precision (91). Thus, some fundamental cellular processes including gene expression, protein localization, and signal transduction have already been commanded by light as a means of external control. To guarantee orthogonality, it is desirable to implement optogenetic systems in biological platforms with null photo-responsiveness. In that context, *S cerevisiae* is a superb blind chassis, where light-sensitive domains from bacteria, filamentous fungi, plants, and animals have been already integrated to gain defined functions without triggering pleiotropic phenotypes (92, 93). Natural or engineered photoreceptors that mainly respond to UV, blue, and red light have been expressed in yeast, allowing the fine control of several processes in clonal populations (92, 93). However, so far, there are scarce reports utilizing optogenetics as an induction strategy in establishing or applying synthetic consortia in yeast. Here, some of these examples are discussed to illustrate how optogenetics lights up the possibility of addressing pending and pressing biological questions, while also eliciting novel and unprecedented behaviors at the community level.

### Optogenetics and spatial dynamics in microbial communities

Unlike classic chemical inducers, optogenetics enables precise stimulation with temporal and spatial control (91). This strategy enables the formation of “pseudo-communities” in cultures, where a subset of a clonal population undergoes significant behavioral changes when illuminated, allowing the study of interactions between subpopulations. Therefore, approaches based on light induction have enormous potential to interrogate biological systems in terms of their spatial organization, as the distribution of cells with distinct phenotypes—but same genotypes—can be precisely controlled (94, 95). Also, localized illumination can induce pattern formation in microbial communities, allowing us to address how intercellular interactions can be locally shaped within a large niche.

**Fig 2:**
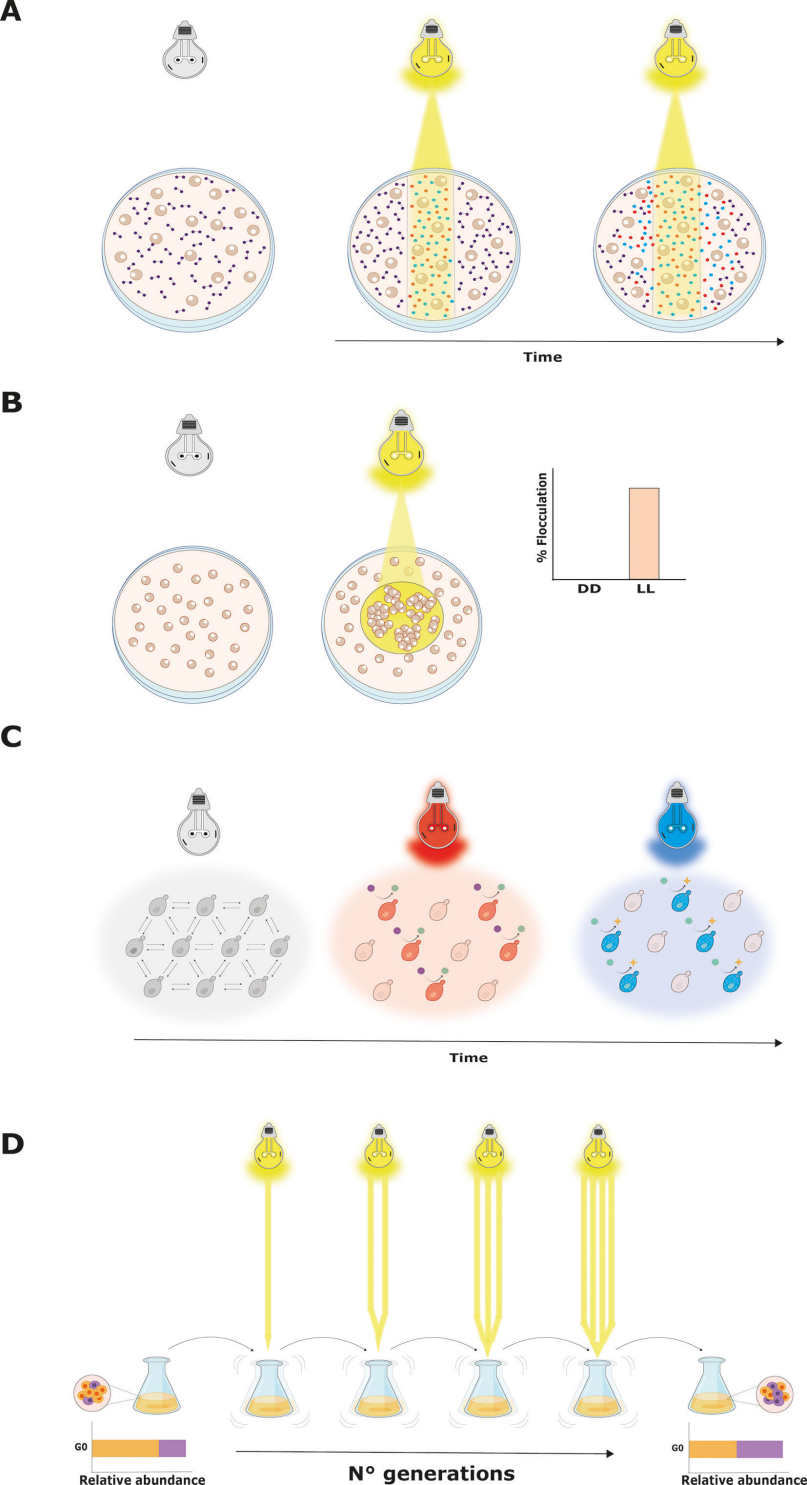
Optogenetic manipulation of yeast communities. Spatial illumination patterns can transform a single clonal population into a pseudo-community by applying specific light arrangements, resulting in marked changes between stimulated and unstimulated cells. This approach allows precise control of metabolic processes, such as disaccharide hydrolysis, within a defined illuminated proportion of cells of a uniform yeast culture. This system allows studying cooperator-cheater dynamics as sugars diffuse over time from an ecological perspective (**A**). Likewise, local delivery of light can change the collective phenotype of a single clonal population, triggering phenomena such as flocculation only in defined parts of the culture (**B**). Moreover, these synthetic systems can be improved by wavelength-specific modulation of metabolic processes in a sequential manner in artificial *S. cerevisiae*-based ecosystems (**C**) or to balance strain ratios in asymmetric co-cultures by increasing light exposure after multiple generations (**D**). Dashed lines represent the illuminated zones where cells are grown in a Petri dish.

Optogenetic approaches have been employed to explore ecological interactions, including phenomena like commensalism. A metabolized or secreted molecule becomes a public good when it can be used for different types of cells in the same medium and not only for those that produced it (96). These diffusible compounds lead to the emergence of cooperative interactions in microbial communities, changing their structure. However, competitive behaviors are also observed in consortia involving public goods production. The latter is due to the appearance of cheater cells—free riders that take advantage of available products without paying the metabolic cost of their biosynthesis (96). In *S. cerevisiae*, a clear example of cooperation-competition dynamics by public goods production is related to the expression of the *SUC2* gene in the presence of sucrose as a carbon source (96). The corresponding sequence encodes for an enzyme with invertase activity that hydrolyzes sucrose in glucose and fructose. To understand how intercellular interactions alter the position and phenotype of cells in a yeast consortium, *SUC2* expression was optogenetically controlled, allowing its expression upon light (97). Thus, using a transcriptional switch based on CRY2-CIB1 heterodimerization, light stimulation allowed cooperators and cheaters to be spatially segregated, forming specific patterns according to the localized cooperation between cells (Fig. 2A). The arrangement of both types of cells is caused by nutrient competition, provoking a discrete ring-like phenotype when growing in Petri dishes (97). The illuminated area displays cooperator saturation, surrounded by a zone of lesser growth and an exterior dense layer of cheaters at the periphery of the plate. It is worth mentioning that the first ring is caused by the inhibition of cheater growth, which is a consequence of the deprivation of other nutrients that the cooperators use. Interestingly, the second ring is formed by the cheaters that manage to reach the hexoses diffused by the action of the cooperators from the center, having more access to the limiting nutrient in the plate. Thus, the spatial arrangement of genotypes within microbial communities determines if the amount of public goods is sufficient for all cells. The production of cooperators needs to be sufficient only to protect themselves from the invasion of cheaters. Thus, when production is low, the cooperators cannot grow (97). However, the cheaters could take over the community if the public good is produced at a high enough concentration. A recent study further explored the spatial distribution of cells with contrasting capacities of hydrolyzing sucrose, adding defined spatial variables to a microbial community, and creating spatially structured landscapes of cheaters and cooperators individuals (98). By using the EL222 optogenetic switch, *SUC2* expression was induced upon light, and depending on the domains specified by the applied light pattern, the pseudo-community behaved as a band-pass filter, where the interaction between cooperator and cheater cells revealed the formation of interesting spatial structures (98). When the production of hexoses from the cooperator cells (specified by the presence of light) was high, the cheaters benefited more and, consequently, cells of the community got closer. Similarly, cooperators benefit more from their own metabolism when cheaters are closer because cooperation mostly occurs at the frontiers of physical interaction. The latter is possible since cheaters also passively cooperate by letting sucrose reach cooperators (98). As the community grows, the absorption rate varies depending on the local population density and the hexose transformation rate. Thus, it is possible to finely define the spatial characteristics of *SUC2* expression, therefore specifying with light the population enabled to behave as cooperators, while cheaters remain in the shadows, providing important insights into how microbial niches are shaped. Such results also highlight the impact of chemical diffusion, consortia composition, and the existing metabolic interdependences, as critical parameters when improved/stable cooperation and efficient productions are aimed at synthetic consortia. Notably, optogenetic control of these and other interactions can incorporate varying photoperiods to regulate the frequency of process activation and deactivation, as well as light gradients to impose precise, gradual patterns of expression/phenotypes.

### Boosting communication by light

Stabilizing a synthetic community over time normally requires effective and productive transmission of information between the different individuals. In that context, a novel system based on light-dependent cell communication was recently described (99). The design consists of two engineered yeast strains, where the first produces αSc by using a FUN-LOV optogenetic switch, whereas the second one can induce a response (quantified as luciferase expression) in the presence of the secreted pheromone. When different strains of both cells, including mutants in downstream signaling of αSc, were co-cultured, the response level depended on the strain genotype and the illumination protocol (99). In addition, the optogenetic intercellular system displayed less basal activity than autonomous strains, confirming that division of labor improves overall performance by noise reduction. Yeast flocculation, a cell aggregation phenotype considered a social behavior under adverse conditions, has also been controlled by light using the same optogenetic switch, FUN-LOV (100). By emulating recent studies, strains whose flocculation capacity is commanded by light are expected to show spatial patterning in the semi-solid medium under specific stimulation regimes (Fig. 2B). On the other hand, extrachromosomal FUN-LOV variants improving the dynamic range of induction have been reported (101). In addition, new FUN-LOV switch versions were recently packed in a single plasmid (FUN-LOV^sp^), including the cloning of antibiotic resistance cassettes to permit their integration at the *HO* locus in order to develop strains of interest: industrial or wild ones (102). Indeed, FUN-LOV^sp^ functionality has already been successfully implemented in a wine yeast strain such as EC1118, extending the switch versatility and making it suitable for bioprocessing settings (102). We expect that these novel optogenetic switches will facilitate the implementation of new synthetic communities, involving different yeast strains.

Another study based on optogenetic control of the yeast mating pathway was recently described (103), where a similar circuit of cell communication to the one described above (99) was utilized but based on the EL222 optogenetic switch. The pheromone gradients were spatially evaluated by delivering light to engineered strains. Combining the optogenetic experiments and simulations from mathematical models, it was possible to learn that the Bar1 protease levels are critical in improving the α-factor sensing by shaping the local concentration in the surroundings of a *MATa* cell (103). On the other hand, a variant of the EL222 optogenetic switch was used to evaluate the influence of communication in the spontaneous emergence of patterning in a yeast culture involving cells with contrasting gene expression levels (104). In the same way, an EL222 light-inducible Cre-LoxP system was also used to create a yeast consortium (105), where a single population can differentiate into two or four states, permitting the formation of a pseudo-community expressing diverse fluorescent reporters. Depending on the molecular design and light treatments, the differentiation rate can be finely tuned to control the composition even in continuous cultures (105). Crucially, and as previously emphasized, these experimental protocols can be significantly enhanced by utilizing spatial light gradients or pulsatile light patterns, enabling high-frequency stimulation. While similar effects could be partially achieved through chemical stimulation and microfluidics, the superior spatial and temporal resolution offered by optogenetic controllers becomes particularly advantageous when applied in solid media.

### Spotlighting unexpected partners

In its infancy, optogenetics mainly focused on using bacterial opsins in human neurons, allowing the rapid and precise turning on or off in the propagation of action potentials (106). The latter is possible because these photoreceptors also behave as ion channels, allowing the transport of different charged molecules (107). Importantly, opsins are broadly distributed in nature and are classified by their different biochemical properties, subcellular localizations, and biological origins. Recently, vacuolar rhodopsin from the filamentous fungus *Ustilago maydis* was implemented in *S. cerevisiae*, allowing its conversion into a facultative photoheterotroph (108). Thus, upon light, the rhodopsin-expressing yeast exhibited a significant increase in fitness under energetically limiting conditions. Considering that yeast cells are heterotrophic, the imposition of light patterns (under nutritional limiting conditions) could lead to the establishment of a pseudo-community where cells with fundamentally distinct types of metabolism interact. In fact, there is already an exciting study showing synthetic consortia involving the budding yeast and a phototrophic organism such as *Chlamydomonas reinhardtii*, where the former converts glucose into CO_2_ which can be photosynthetically fixed by the latter (109). In parallel, the green algae supplies ammonium from nitrite for *S. cerevisiae* growth, allowing overall community maintenance by establishing a mutualistic interaction (109). On the other hand, it has become evident that optogenetics also represents a potent strategy to control metabolic engineering (110, 111). In that context, a pioneering study has shown the successful combination of bioprocessing, light induction, and interspecies co-cultures (112). Yeast and *E. coli* strains were engineered to establish a community, where the bacterium growth was finely controlled by illumination through a switch modulating a toxin-antitoxin system. Upon determined light conditions, the composition was tuned to improve the production of isobutyl acetate and the flavonoid naringenin. Specifically, the production of these valuable metabolites was optimized through a division of labor, with *S. cerevisiae* utilizing intermediates synthesized by the bacteria (112).

### Advancing reproducibility

Another critical aspect in generating synthetic systems such as microbial communities is reproducibility and the ability to technically implement defined culture settings. In the past years, there has been a remarkable improvement in the technology designed to deliver light stimulation. Currently, these devices are designed for full automatization in terms of temporality, spatial arrangement, intensity, and the wavelengths of the lighting conditions. Furthermore, many of them are based on open-source codes and software, allowing the globalization of their application and easy implementation in teaching settings. Consequently, results obtained based on optogenetic approaches can be more reproducible across laboratories. Examples, such as LUSTRO (113), Diya (114), and OptoCube (98), have demonstrated that optogenetics can be applied to cell cultures in different formats, including petri dishes and microplates of varying capacities.

### The rise of optoecology: toward a bright future

In this way, coupling light-induction and synthetic yeast consortia could facilitate the implementation of spectral multiplexing, i.e., the application of different wavelengths for multi-chromatic control of diverse biological processes without interference (115) (Fig. 2C).

Relevantly, another opportunity awaiting to be fully exploited is the combination of optogenetics and experimental evolution in yeast consortia, where engineered strains could generate synergic interactions after a high number of culture passages exposed to periodic stressful illumination treatments (Fig. 2D) (116). Such approaches will permit combining ecologically relevant questions with the power of experimental evolution. Indeed, recent work, such as the development of “optovolution” in yeast cells, demonstrates how optogenetics can be integrated into evolution paradigms to optimize protein functionality (116). While such efforts are still at their infancy, they can pave the way for their integration into synthetic microbial communities for evolving defined interspecies interactions and resource sharing.

In summary, mounting evidence highlights how optogenetic approaches enable the study of community-level processes in unprecedented ways. Thus, we are now witnessing the dawn of the “optoecology” era, with *S. cerevisiae* synthetic communities taking the spotlight.

## CONCLUDING REMARKS

Since life takes place and evolves in diverse environments, synthetic communities are revolutionizing how biological agencies can be studied as part of complex ecosystems. These artificial consortia show favorable aspects over monocultures such as modularity and extended metabolic capabilities. This review mainly concentrated on the experiences involving *S. cerevisiae* communities, focusing on synthetic ecology, bioprocessing, and biosensing. In recent years, optogenetics has already emerged as a promising induction strategy to evaluate how external and controlled perturbations could alter the behavior of a whole ecosystem. However, light induction still represents a strategy with an underexplored potential to modulate biology at a spatial scale. Combining optogenetics and synthetic consortia will allow researchers to improve the capacity to generate community-level patterns and behaviors relevant to fundamental and applied processes.
